# Short Wavelength Automated Perimetry, Standard Automated Perimetry, and Optical Coherence Tomography in Dominant Optic Atrophy

**DOI:** 10.3390/jcm13071971

**Published:** 2024-03-28

**Authors:** Marco Lombardo, Andrea Cusumano, Raffaele Mancino, Francesco Aiello, Roberto Pietro Sorge, Carlo Nucci, Massimo Cesareo

**Affiliations:** 1 Ophthalmology Unit, Department of Experimental Medicine, University of Rome Tor Vergata, 00133 Rome, Italy; 2 Laboratory of Biometry, Department of Systems Medicine, University of Rome Tor Vergata, 00133 Rome, Italy

**Keywords:** optic neuropathies, ADOA, autosomal dominant optic atrophy, SWAP, short wavelength automated perimetry, OCT, optical coherence tomography

## Abstract

**Background:** Blue-yellow axis dyschromatopsia is well-known in Autosomal Dominant Optic Atrophy (ADOA) patients, but there were no data on the correlation between retinal structure and short-wavelength automated perimetry (SWAP) values in this pathology. **Methods:** In this cross-sectional case-control study, we assessed the correlation between best corrected visual acuity (BCVA), standard automated perimetry (SAP), SWAP, and optical coherence tomography (OCT) parameters of 9 ADOA patients compared with healthy controls. Correlation analysis was performed between BCVA, mean deviation, pattern standard deviation (PSD), and fovea sensitivity (FS) values and the OCT thickness of each retinal layer and the peripapillary retinal nerve fiber layer (pRNFL). **Results:** The following significant and strong correlations were found: between BCVA and ganglion cell layer (GCL) and the global (G) pRNFL thicknesses; between SAP FS and GCL and the G-pRNFL thicknesses; between SWAP PSD and total retina, GCL, inner plexiform layer, inner nuclear layer, inner retinal layer and the temporal pRNFL thicknesses. We found a constant shorter duration of the SITA-SWAP compared with the SITA-STANDARD strategy. **Conclusions:** SWAP, SAP, and BCVA values provided relevant clinical information about retinal involvement in our ADOA patients. The perimetric functional parameters that seemed to correlate better with structure involvement were FS on SAP and PSD on SWAP.

## 1. Introduction

Autosomal dominant optic atrophy (ADOA) is the most common hereditary optic neuropathy, with a prevalence of 1/10,000 to 1/30,000 in the world [1].

Mutations in the *OPA1* gene cause 57 to 89% of ADOA cases. This gene encodes the mitochondrial dynamin-related GTPases in the inner mitochondrial membrane and is involved in many mitochondrial activities. In particular, the *OPA1* protein plays a crucial role in regulating mitochondrial fusion and apoptosis by modulating the inner mitochondrial membrane [1]. To date, more than 400 *OPA1* mutations have been reported. Of the reported pathogenic *OPA1* variants, 28% are missense variants, 24% are classified to induce aberrant splicing, 22% are frameshift variants, 15% are nonsense variants, and 7% are structural variants [2].

The resulting mitochondrial dysfunction mostly affects retinal ganglion cells, probably due to some peculiar characteristics of this type of cell [3].

The first clinical manifestations begin gradually from infancy and include a variable reduction of visual acuity, sometimes asymmetrically; temporal or diffuse pallor or excavation of the optic disc, and centro-cecal or central scotoma in visual field testing. Since there is a remarkable phenotypic heterogeneity even within the same family, the final visual acuity can vary from a slight decrease to legal blindness [4,5,6,7].

Another peculiar feature of the disease is an altered color perception, particularly dyschromatopsia on the blue-yellow axis, demonstrated by color perception tests: tritanopia is the typical defect found at the Farnsworth-Munsell (FM) 100-hue test and seems to be one of the most sensitive indicators of ADOA along with optic disc pallor [5,8].

Visual field tests have long been used in clinical ophthalmology and medical practice in general to determine the differential light sensitivity corresponding to the visual field examined. Testing of multiple retinal locations allows the detection and classification of visual abnormalities ranging from a general depression of sensitivity to the identification of specific regions of sensitivity loss [9]. Its ability to provide this important clinical information has allowed the diffusion of the method and its subsequent validation as an ophthalmic diagnostic tool to evaluate, not only the function of the retina and the optic nerve, but of the entire visual pathway.

White-on-white or standard automated perimetry (SAP) has become a common test for diagnosing and monitoring all types of optic neuropathies [10].

Short-wavelength automated perimetry (SWAP) presents a blue stimulus to selectively stimulate the blue cones. It also uses a high-luminance yellow background to saturate the red and green cones and the rods [11].

It was designed to find a method to diagnose early initial damage of ganglion cells, particularly in glaucoma, but this type of perimetry has been shown to have problems that have limited the diffusion of this method. It has a greater variability associated with the estimation of the threshold, absorption of the stimulus by ocular media, increase in the duration of the exam, and an additional learning effect [11]; the latter two were largely resolved with the advent of the Swedish interactive thresholding algorithm (SITA) strategy [12].

In 2006 Walters et al. published a case study on 5 patients affected by ADOA in which they found a clear difference between the mean values of mean deviation (MD) at SAP versus SWAP. MD values at SWAP were on average 11.46 decibels (dB) lower than at SAP. The authors suggested that this difference may help diagnose ADOA both in the early and advanced phases [13].

Optical coherence tomography (OCT) is a non-invasive diagnostic imaging technique. It allows for microscopic visualization and analysis of retinal structures by tomographic sections of the retina. It is based on the interference between the signal of an object under investigation and a local reference signal. OCT can produce a cross-sectional image of the object in real time, i.e., a two-dimensional image in space using confocal optics. In this way, both lateral and axial resolution are determined by the numerical aperture of the microscope objective. In OCT, the axial resolution is mainly determined by the optical source and therefore the retina of the human eye can be acquired with a very high axial resolution [14]. This technology has had extensive development not only in the medical field but also in the industrial and agricultural fields [15]. It has become part of clinical practice in ophthalmology thanks to its ease and speed of execution and its application has spread not only to retinal diseases, but also to those of the optic nerve and neurological or systemic diseases.

Numerous works have studied ADOA using spectral-domain OCT, showing a reduction in the thickness of the inner retina of patients, due to the ganglion cell layer involvement at the posterior pole and the reduction of the peripapillary retinal nerve fiber layer (pRNFL) thickness, especially in the temporal sector [16,17].

On the contrary, there are few works about the correlation between the structural parameters measured by OCT and the parameters expressing the function of the visual system; some of the functional parameters tested were visual acuity, electro-functional neurophysiological exams, and differential light sensitivity data measured at microperimetry [18,19,20,21,22].

This study aims to assess the correlation between SAP, SWAP and OCT parameters of patients with ADOA genetically confirmed by the presence of the *OPA1* gene mutation. Given the typical dyschromatopsia on the blue-yellow axis and the preliminary results of the case series by Walters et al. [13], the hypothesis was that SWAP can also provide important information on retinal structural involvement in patients affected by ADOA.

## 2. Materials and Methods

This cross-sectional, case-control, observational study included 9 patients (5 males and 4 females) from 3 unrelated families with a genetically established diagnosis of ADOA caused by the *OPA1* gene mutation. They were enrolled and examined at the Regional Reference Center for Low Vision and Visual Rehabilitation at the University of Rome Tor Vergata between April 2021 and April 2022 and were compared with 9 healthy controls (HCs), age and sex-matched, randomly enrolled in our general clinic.

Informed consent was obtained from all participants in accordance with the Declaration of Helsinki. The study was approved by the internal review board of the University of Tor Vergata in Rome (ethics approval ID: 265.21).

Genetic counseling was offered to all patients and their families. 7 patients had exon 29 mutation with the nucleotide change c.2873_2876del and the amino acid change p.(Val958Glyfs*3). One patient had a mutation at exon 8 with c.815T>C and p.L272P amino acid change and one patient at exon 19 with C.1870 of G nucleotide change (splicing defect). All these mutations were registered in the *OPA1* mutations database [23].

**Figure 1:**
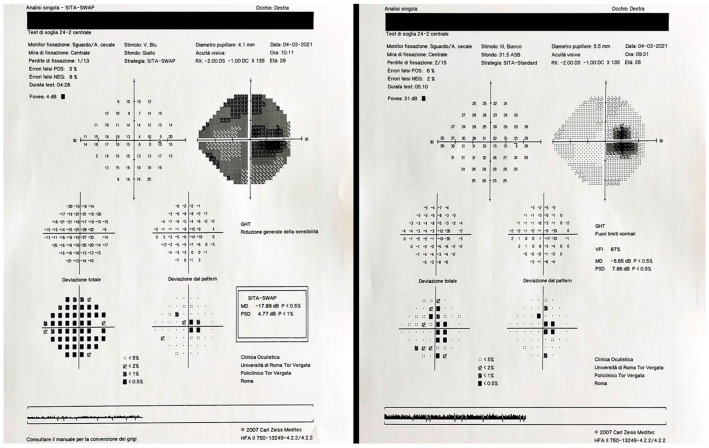
Short-wavelength automated perimetry with SITA-SWAP program 24−2 (**left**) and standard automated perimetry with SITA-Standard program 24−2 (**right**) of the right eye of the same patient affected by autosomal dominant optic atrophy performed with a Humphrey Field Analyzer.

All patients and HCs underwent a comprehensive ophthalmologic examination including best corrected visual acuity (BCVA) measurement with standardized ETDRS tables; FM 100 Hue test; SAP with SITA-Standard program 24-2 and SWAP with SITA-SWAP program 24-2 of a Humphrey Field Analyzer in randomized order (Figure 1); Goldmann applanation tonometry; slit-lamp biomicroscopy with dilated fundus examination with assessment of lens opacity by *Lens Opacities Classification System III* (LOCS III) grading. All participants underwent the “Posterior Pole” scanning protocol of SD-OCT (Spectralis; Heidelberg Engineering, Heidelberg, Germany) to obtain the following automatic layer segmentation: total retinal (RETINA); retinal nerve fiber layer (RNFL); ganglion cell layer (GCL); inner plexiform layer (IPL); inner nuclear layer (INL); outer plexiform layer (OPL); outer nuclear layer (ONL); retinal pigment epithelium (RPE); inner retinal layers (IRL) and outer retinal layers (ORL). Within the “Posterior Pole” scan, macular ETDRS grid data were acquired for each retinal layer in the following sectors: center (C); inner-temporal (ITEM); inner-superior (ISUP); inner-nasal (INAS); inner-inferior (IINF); outer-temporal (OTEM); outer-superior (OSUP); outer-nasal (ONAS); outer-inferior (OINF). The average values of the aforementioned sectors were also calculated. Peripapillary RNFL thickness (pRNFL) was also measured in all subjects using the peripapillary RNFL scanning protocol (3.5 mm diameter). The following thickness values were obtained: global (G); temporal (T); temporal-superior (TS); nasal-superior (NS); nasal (N); nasal-inferior (NI); temporal-inferior (TI); papillo-macular bundle (PMB); N/T. All SD-OCT scans were acquired by the same experienced operator after pharmacologic mydriasis. Only scans with signal quality > 25 were included, while scans with altered segmentation, insufficient illumination or the presence of artifacts were excluded. No manual segmentation correction was required. Exclusion criteria were the presence of retinal and/or optic nerve pathology excluding ADOA, previous intraocular surgery except for uncomplicated phacoemulsification with intraocular lens implantation, current use of any drug therapy known to be toxic to the retina and/or optic nerve, cylindrical refractive errors greater than 2 diopters, spherical refractive errors greater ± 3 diopters; patients with lens opacity that could alter blue-yellow visual field results were also excluded. Regarding the perimetry tests, they were almost all found to be reliable (false positive or negative errors < 15%; fixation losses < 3) probably because all patients were familiar with performing these tests routinely every year. Only one eye of a patient was excluded due to poor fixation ability. Global indices, including the mean deviation (MD) and pattern standard deviation (PSD) of each perimetry, were registered. Foveal sensitivity (FS) values were also registered. Inclusion criteria for the control group were: BCVA of at least 0.0 logMAR, spherical or cylindrical refractive errors lower than 3 and 2 diopters, respectively, normal intraocular pressure (<21 mmHg), normal optic disc appearance, no significant ocular diseases, and no family history of glaucoma or systemic disease with possible ocular involvement. The evaluation methods were comparable between cases and HCs since both groups, who were homogeneous in terms of sex and age, underwent the same tests without requiring any manual corrections by the operators.

The study’s primary outcome was to evaluate the correlation between the functional parameters obtained by the SWAP and SAP perimetries and the structural parameters of the retinal thicknesses measured by OCT in patients affected by ADOA. Secondary outcomes included evaluating whether perimetric indices can differentiate ADOA patients from HCs and determining if SWAP perimetry could be the preferred perimetry method for this population.

### Statistical Analysis

All data were initially entered into an Excel spreadsheet (Microsoft, Redmond, MA, USA) and analysis was performed using the statistical package for the social sciences Windows, version 15.0 (SPSS, Chicago, IL, USA). Descriptive statistics consisted of mean ± standard deviation for the parameters with normal distributions (after confirmation with histograms and Kolgomorov-Smirnov test), median and range (minimum and maximum) for variables with non-normal distributions. Comparison of normal variables between groups (ADOA vs. HCs) was performed with Anova one-way test while the proportions of occurrences were tested by the chi-square test. AUROC (area under receiver operating characteristics) curve analysis was used to identify the indices of the two perimetry exams with the best ability to discriminate between ADOA patients and HCs. ROC curve analysis provided cut-off values for all parameters. The possible linear correlation between the structural parameters (provided by the SD-OCT) and the functional parameters (MD, PSD, FS of each perimetry test and BCVA), was calculated using Pearson’s linear correlation coefficient (r). The *p*-value < 0.05 was considered as the threshold for statistical significance.

## 3. Results

All patients and HCs who met the inclusion and exclusion criteria were confirmed eligible for the present study. A total of 17 eyes of 9 patients (average age 42.55 ± 15.94 years, range: 28–72 years) with a diagnosis of ADOA genetically confirmed by mutation of the *OPA1* gene were analyzed. They were compared with 17 eyes of 9 HCs (average age 43.11 ± 16.75 years, range: 27–74 years). The characteristics of patients, including lens opacity evaluations by LOCS III classification, are summarized in Table 1.

One patient and one HC were pseudophakic with clear lens implantation in both eyes. Regarding ADOA patients, the average BCVA was 0.44 ± 0.35 logMAR (range: 0.0–1.3). The mean duration of the SAP was 6 min and 20 s while it was 4 min and 52 s for the SWAP with all the latter examinations taking less time than the SAP exams (*p* < 0.001). HCs also took less time in performing perimetry with the SITA-SWAP than with the SITA-Standard strategy. Absolute MD values were on average higher at SWAP (−15.32 dB) than at SAP (−5.63 dB), while PSD values were on average higher at SAP (5.62) than at SWAP (4.58). FS values were on average more markedly reduced at SWAP (10.94 dB) compared to SAP (29.47 dB). The distribution of all values investigated was normal, allowing the possible linear correlation between the parameters to be calculated using Pearson’s coefficient.

The following significant (*p* ≤ 0.001) correlations were found:

A strong negative correlation between BCVA values (measured in logMAR unit) and average GCL thickness (r = −0.784) on the ETDRS grid;
A strong negative correlation between BCVA values and the G-pRNFL thickness (r = −0.740);
A strong positive correlation between SAP FS and average GCL thickness (r = 0.749) on the ETDRS grid;
A strong positive correlation between SAP FS and the G-pRNFL thickness (r = 0.707);
Strong negative correlations between SWAP PSD and RETINA (r = −0.726), GCL (r = −0.702), IPL (r = −0.705), INL (r = −0.766), IRL (r = −0.700) average thicknesses on the ETDRS grid;
A strong negative correlation between SWAP PSD and the T-pRNFL thickness (r = −0.736).

All correlations between the functional parameters and the mentioned retinal layers and their significance levels are reported in Table 2.

The use of ROC curves using OCT parameters has already been shown to be useful in this type of patient in previous works [22,24]. We decided to use this type of analysis also for the values obtained from the two functional tests.

**Figure 2:**
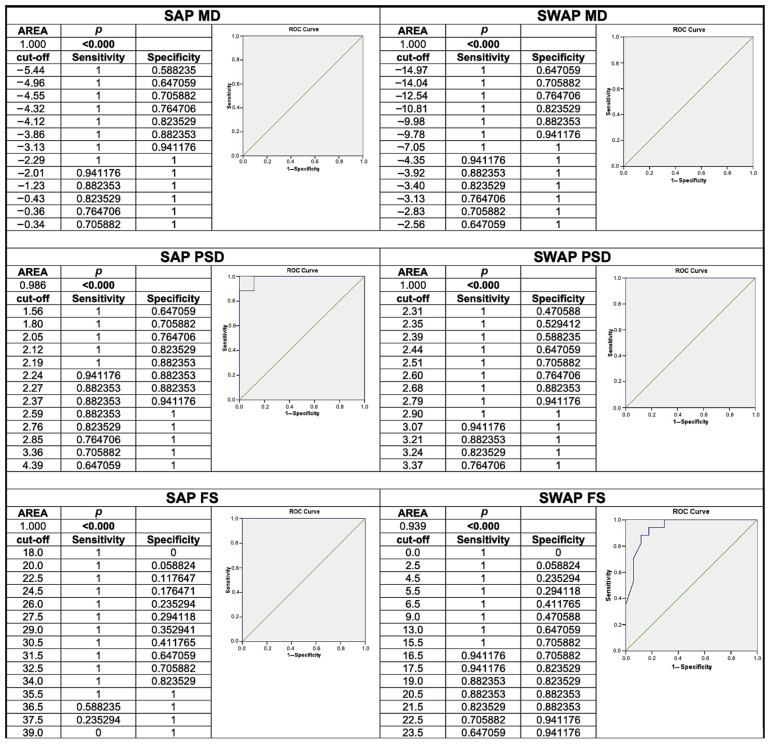
ROC curves of the mean deviation (MD), pattern standard deviation (PSD), and foveal sensitivity (FS) of standard automated perimetry (SAP) and short-wavelength automated perimetry (SWAP); *p*: level of significance (*p*-value).

The analysis of the ROC curves revealed that the parameters of both perimetry tests could well distinguish ADOA patients from HCs. SAP MD and FS along with SWAP MD and PSD showed an area under the curve of 1.000. SAP PSD and SAP FS showed an area under the curve of 0.986 and 0.939, respectively. ROC curves are shown in Figure 2.

## 4. Discussion

The study of the visual field in ADOA patients has always been clinically relevant since the earliest definitions of the pathology [25]. Another fundamental feature of this disease was the temporal involvement of the optic nerve; later studies with OCT revealed that not only the PMB is involved, but all the peripapillary nerve fibers and the ganglion cell population at the posterior pole [22,26]. These features, together with a typical dyschromatopsia in the blue-yellow axis, make SAP, SWAP, microperimetry, and OCT ideal for the morphological and functional assessment of this pathology. In addition, the generalized reduction in retinal sensitivity due to cataracts that typically influences perimetry results, in particular SWAP, is less relevant in this type of patient given the young age at diagnosis [27].

**Figure 3:**
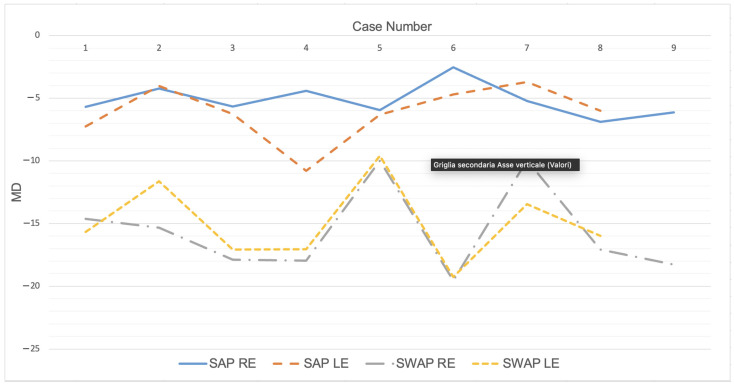
Graphical representation of mean deviation (MD) of each autosomal dominant optic atrophy patient measured at standard automated perimetry (SAP) and short-wavelength automated perimetry (SWAP) showing the difference between the two tests. RE: right eye; LE: left eye.

Only one work to date has evaluated both types of perimetry in the same ADOA patients, demonstrating the usefulness of SWAP in this disease [13]. In agreement with this study, despite the different threshold strategies, we found a 9.69 dB separation between the mean MD data of the two perimetries, with the SWAP MD consistently reduced more than that of SAP in each patient (*p* < 0.001) (Figure 3). This finding could strengthen the hypothesis that this difference may be an indicator of ADOA.

To date, no study tried to correlate SWAP values with structural data measured with OCT. We found significant correlations between the MD, PSD and FS values of both perimetry exams and the thickness values measured by OCT. Each type of perimetry was found to correlate better with specific morphological values on OCT, suggesting that both examinations are useful to investigate different aspects of the same pathology.

One of the strongest and most significant correlations found was between the PSD at the SWAP and the T value of the pRNFL thickness. The correlation proved to be negative, meaning that an increase in PSD corresponds to a decrease in OCT values. Since the reduction in differential light sensitivity in these patients was shown to be greater at the level of the papillomacular bundle (centrocecal scotoma) than at other points, we can assume that the PSD value increases in relation to the greater involvement of this bundle than the surrounding retina. The PSD value at the SWAP also showed a strong negative correlation with the thicknesses of RETINA, GCL, IPL, INL, and IRL of the ETDRS grid. The thickness of the inner retinal layers is typically reduced in this disease, not only at the level of the papillomacular bundle, but in all the posterior pole, especially in more advanced stages. This appears to be caused by a primary involvement of the more damage-sensitive small fibers of the parvocellular and koniocellular nuclei and by the subsequent damage of the residual larger ganglion cells [17]. Only moderate and not significant (except for the T-pRNFL value) correlations were found between the PSD at the SAP and the same OCT values mentioned above both in the ETDRS grid and in the peripapillary zone. A possible explanation for the better correlation of SWAP compared to SAP may result from the more selective stimulus-response of the blue cones and thus the corresponding koniocellular ganglion cells, which seem to be more sensitive to damage along with parvocellular cells [2]. In the case of the standard stimulus, cones and rods are stimulated, and therefore every type of ganglion cell.

A strong positive correlation was found between the FS value at the SAP and the thickness of the GCL layer of the ETDRS grid. The same strong correlation was also found with the G-pRNFL thickness. This may be due to the higher concentration of photoreceptors at the foveal level, which transduces the signal to the same conspicuous number of ganglion cells whose axons go to form the pRNFL. Primary damage to these ganglion cells can progressively lead to a reduction in nerve fibers and a consequent decrease in the sensitivity of the corresponding photoreceptors at the foveal level.

A slightly higher correlation coefficient also emerged between BCVA values and the aforementioned OCT thicknesses; this is because, as expected, SAP FS correlates strongly in turn with BCVA (r = −0.740; *p* < 0.001). A possible explanation for the absence of correlation between FS at the SWAP and OCT values may be due to the almost complete absence of blue cones at the level of the fovea [28,29].

No correlations were found between the parameters of the two perimetries and the RNFL value on the ETDRS grid. We can therefore conclude that regarding RNFL thickness, the peripapillary zone has a better functional correlation than the posterior pole because pRNFL thickness includes all the retinal ganglion cell fibers instead of those from only the posterior pole.

Regarding the ability of SAP and SWAP to distinguish between ADOA patients and HCs, all perimeter indices considered showed excellent sensitivity and specificity. MD values of both perimetry tests showed an AUROC equal to 1, indicating that these patients, both for SAP and, as previously mentioned, even more so for SWAP, have a diffuse light sensitivity defect very different from HCs. PSD value showed a greater area under the ROC curve for SWAP, while the area of FS was greater in SAP. This finding could precisely reflect the greater structural correlation of these parameters.

Performing two types of perimetry in ADOA patients can be difficult in routine clinical practice; since the BCVA assessment is a simpler, standardized and faster procedure than a perimetry exam, and provides better correlations with structural parameters than SAP, we suggest, for the assessment of functional response to structural involvement, the possibility of performing only SWAP in ADOA patients without significant lens opacity, along with visual acuity measurement, which remains a routine practice in ophthalmologic examinations. Another evidence in favor of this possibility is that, in our cohort of patients and HCs, the test durations with the SITA-SWAP strategy were consistently shorter than that with the SITA-SAP strategy. To the best of our knowledge, this is the first study to emphasize the fact that, with the SITA strategy, the duration of the SWAP perimetry is shorter than that of the SAP. This data could partially solve the problem of the “fatigue effect”. This perimetry also showed a more depressed MD than at SAP in ADOA patients. The problem of variability in threshold estimation described for SWAP, in our cohort of patients, did not affect distinguishing them from controls. On the other hand, the influence of long-term fluctuation could not be assessed in this study, as this will require repeated examination in the future. Even the low MD values at SWAP may limit the ability to detect changes in time.

## 5. Conclusions

Both SAP and SWAP could provide relevant clinical information on retinal involvement in our cohort of ADOA patients. The most important functional parameters that seemed to correlate better with structure involvement were FS on SAP and PSD on SWAP. The main limitations of this study were the retrospective nature, the limited number of patients and the comparison of two perimetry tests, which, even if performed correctly by the same patient, still have different dynamic ranges, normative values and variability characteristics. Further studies with larger numbers of patients with ADOA will be needed to corroborate these findings and define which functional examination may be the most suitable, not only for studying current structural involvement, but also for monitoring disease progression.

## Figures and Tables

**Table 1:** Demographic and clinical characteristics of autosomal dominant optic atrophy patients enrolled in the present study.

Case Number	Sex	Age Range (Years)	Eye	BCVA (logMAR)	LOCS III
1	M	25–30	R	0.5	N = 1; C = 1; P = 1
			L	0.4	N = 1; C = 1; P = 1
2	M	25–30	R	0.5	N = 1; C = 1; P = 1
			L	0.5	N = 1; C = 1; P = 1
3	M	30–35	R	0.1	N = 1; C = 1; P = 1
			L	0.0	N = 1; C = 1; P = 1
4	M	30–35	R	0.5	N = 1; C = 1; P = 1
			L	0.5	N = 1; C = 1; P = 1
5	M	55–60	R	1.0	N = 2; C = 1; P = 1
			L	0.7	N = 2; C = 1; P = 1
6	F	25–30	R	0.5	N = 1; C = 1; P = 1
			L	0.5	N = 1; C = 1; P = 1
7	F	50–55	R	0.1	N = 2; C = 1; P = 1
			L	0.1	N = 2; C = 1; P = 1
8	F	50–55	R	1.3	N = 2; C = 1; P = 1
9	F	70–75	R	0.0	pseudophakia
			L	0.2	pseudophakia

M: male; F: female; R: right; L: left; BCVA: best corrected visual acuity; LOCS III: lens opacities classification system III.

**Table 2:** Pearson’s correlation coefficients and their significance levels between optical coherence tomography thicknesses and functional parameters in our cohort of autosomal dominant optic atrophy patients. Significant correlations are highlighted in bold type.

		MD SAP	MD SWAP	PSD SAP	PSD SWAP	FS SAP	FS SWAP	BCVA
G-pRNFL	r	0.00232	0.10835	−0.40134	**−0.55558**	**0.70773**	−0.06758	**−0.74007**
	*p*	0.99296	0.67891	0.11033	**0.02059**	**0.00148**	0.79664	**0.00068**
T-pRNFL	r	−0.00967	−0.31899	**−0.53518**	**−0.73675**	0.45797	−0.34587	**−0.53818**
	*p*	0.97062	0.21205	**0.02684**	**0.00074**	0.06451	0.17388	**0.02584**
Average RETINA	r	−0.11448	−0.21884	−0.35899	**−0.72688**	**0.56497**	−0.21187	**−0.62560**
	*p*	0.66174	0.39874	0.15703	**0.00095**	**0.01812**	0.41429	**0.00723**
Average GCL	r	−0.04230	−0.08871	−0.39781	**−0.70236**	**0.74976**	0.15548	**−0.78435**
	*p*	0.87194	0.73494	0.11379	**0.00167**	**0.00053**	0.55126	**0.00019**
Average IPL	r	−0.15004	0.03093	−0.46004	**−0.70537**	**0.54795**	−0.07439	**−0.66833**
	*p*	0.56544	0.90618	0.06316	**0.00156**	**0.02278**	0.77661	**0.00336**
Average INL	r	−0.05794	−0.22725	−0.45809	**−0.76630**	**0.59467**	0.00954	**−0.60471**
	*p*	0.82519	0.38039	0.06443	**0.00033**	**0.01181**	0.97101	**0.01013**
Average IRLs	r	−0.21088	0.00596	−0.24905	**−0.70039**	0.29028	0.06938	**−0.61769**
	*p*	0.41654	0.98188	0.33507	**0.00174**	0.25837	0.79134	**0.00824**

MD: mean deviation; PSD: pattern standard deviation; FS: fovea sensitivity; SAP: standard automated perimetry; SWAP: short-wavelength automated perimetry; BCVA: best corrected visual acuity; G-pRNFL: global peripapillary retinal nerve fiber layer; T-pRNFL: temporal peripapillary retinal nerve fiber layer; GCL: ganglion cell layer; IPL: inner plexiform layer; INL: inner nuclear layer; IRLs: inner retinal layers; r: Pearson’s correlation coefficient; *p*: significance level.

## Data Availability

The datasets generated during the current study are available from the corresponding author on reasonable request.
